# DTL promotes cancer progression by PDCD4 ubiquitin-dependent degradation

**DOI:** 10.1186/s13046-019-1358-x

**Published:** 2019-08-13

**Authors:** Haoran Cui, Qin Wang, Zhenchuan Lei, Maoxiao Feng, Zhongxi Zhao, Yunshan Wang, Guangwei Wei

**Affiliations:** 1 Department of Cell Biology and Key Laboratory of Experimental Teratology, Ministry of Education, Shandong University School of Medicine, Jinan, Shandong China; 2 Department of Anesthesiology, Qilu Hospital, Shandong University, Jinan, Shandong China; 3 College of Marine Life Sciences, Ocean University of China, Qingdao, Shandong China; 4 School of Pharmaceutical Sciences, Shandong University, No. 44 West Wenhua Road, Jinan, Shandong China; 5 Department of Clinical Laboratory, The Second Hospital of Shandong University, Jinan, Shandong China

**Keywords:** DTL, CUL4A, PDCD4, Cancer development

## Abstract

**Background:**

Ubiquitin E3 ligase CUL4A plays important oncogenic roles in the development of cancers. DTL, one of the CUL4-DDB1 associated factors (DCAFs), may involve in the process of cancer development. Programmed cell death 4 (PDCD4) is a tumor suppressor gene involved in cell apoptosis, transformation, invasion and tumor progression.

**Methods:**

Affinity-purification mass spectrometry was used to identify potential DTL interaction proteins. Co-immunoprecipitation (Co-IP) was performed to verify protein interaction between DTL and PDCD4. mRNA levels in cancer cells and tissues were detected by Quantitative real-time PCR. Lentivirus was used to establish stable overexpression and knocking down cell lines for DTL and PDCD4. Transwell and wound healing assays were used to determine migration ability of cancer cells. Matrigel assay was used to determine invasion ability of cancer cells. MTT and colony formation assays were used to evaluate proliferation of cancer cells.

**Results:**

In this study, programmed cell death 4 (PDCD4) was identified as a potential substrate of DTL. Co-IP and immunofluorescence assays further confirmed the interaction between DTL and PDCD4. Moreover, DTL overexpression decreased the protein level and accelerated the degradation rate of PDCD4. Through in vitro ubiquitination experiment, we proved that PDCD4 was degraded by DTL through ubiquitination. Clinically DTL was significantly up-regulated in cancer tissues than that in normal tissues. The survival curves showed that cancer patients with higher DTL expression owned lower survival rate. Functional experiments showed that DTL not only enhanced the proliferation and migration abilities of cancer cells, but also promoted the tumorigenesis in nude mice. Rescued experiment results demonstrated that silencing PDCD4 simultaneous with DTL recovered the phenotypes defect caused by DTL knocking down.

**Conclusions:**

Our results elucidated that DTL enhanced the motility and proliferation of cancer cells through degrading PDCD4 to promote the development of cancers.

**Electronic supplementary material:**

The online version of this article (10.1186/s13046-019-1358-x) contains supplementary material, which is available to authorized users.

## Background

The ubiquitin-proteasome proteolysis system responsible for degrading proteins is involved in nearly all cellular processes [1]. Dysregulation in the components of the ubiquitin system leads to a variety of diseases such as cancers [2, 3]. Cullin protein family containing conserved cullin homology domain is a major type of E3 ligase [4, 5]. CUL4A (Cullin 4A), a member of the cullin protein family, forms CRL4A (cullin 4A–RING ubiquitin ligase) complex with a ring finger protein ROC1, an adapter protein DDB1, and DDB1 cullin4 associated factors (DCAFs) [6].

In CRL4A complex, DCAFs act as substrate recognizers to mediate the substrate degradation directly. DTL, also called CDT2, DCAF2 or RAMP, is one of the DCAFs containing seven WD40 domains in its N-terminal. DTL is first identified as retinoic acid-regulated nuclear matrix-associated protein (RAMP) since it is down-regulated during RA-induced differentiation of NT2 cells [7]. The roles of DTL in regulating DNA replication and cell cycle were well-illustrated so far [8]. For example, studies in yeast revealed that DTL deletion severely slowed down S-phase progression [9]. DTL substrates, such as Cdt1, PR-Set7/Set8 and p21, render DTL preventing cells from DNA damage in S phase and after UV irradiation [10–15].

The essential roles of DTL in genomic stability suggest that this ubiquitin system component may involve in tumorigenesis [16]. Remarkably, previous studies showed that DTL expression was elevated in many cancers [17–20]. Previously studies in breast cancer and Ewing sarcoma showed that DTL knockdown weakened the proliferation and migration abilities of cancer cells [17, 19]. However, the systematic studies on DTL functions in cancers remains to be evaluated. More importantly, the underlying mechanisms of DTL regulating cancer progression need more investigation.

Programmed cell death 4 (PDCD4) is a tumor suppressor gene involved in cell apoptosis, transformation, invasion, and tumor progression [21, 22]. PDCD4 exerts its activities by interacting with eukaryotic translation initiation factors 4A (eIF4A) and 4G (eIF4G) [23, 24]. It has been found that PDCD4 expression deficiency was closely associated with the progression and prognosis of various cancers, such as the myloid leukemia, lung, ovarian, colon and glima cancers [25–29]. Considering the importance roles of PDCD4 in cancer progression, the regulation of PDCD4 level in cancers deserves more investigation.

In our study, a novel PDCD4 degradation way via ubiquitin system was elucidated. The results of this study suggested that DTL bound with and down-regulated PDCD4 by ubiquitin degradation and promoted the migration, invasion and proliferation abilities of cancer cells. Above all, our data provided novel mechanistic insights into the function of DTL in cancer progression.

## Material and methods

### Cell lines and cell culture

MCF7, MDA-MB-468 and 293 T cells were preserved by our laboratory and cultured in DMEM with 10% FBS. MDA-MB-231, BT549, SKBR-3, H1650 and A549 cells were preserved by our laboratory and cultured in RPMI-1640 medium with 10% FBS. Human breast epithelial cell line MCF-10A was cultured in DMEM/F12 with 5% horse serum (Biological Industries), 10 μg/ml insulin (Solarbio), 20 ng/ml epidermal growth factor (Pepro Tech), 100 ng/ml cholera toxin (Macgene), 0.5 μg/ml hydrocortisone (Macgene). All cells were grown at 37 °C with 5% CO_2_/95% air atmosphere and were revived every 3 to 4 months.

### Plasmid constructions

Wide type DTL and truncated DTL cDNA sequences were constructed into PLVX-AcGFP-N1 vector. shRNA sequences for DTL and PDCD4 were constructed into pLKO.1-puro vector. shRNA sequences used to establish a conditional MDA-MB-231 DTL silent cell line were constructed into Tet-pLKO-puro plasmid. All the plasmids were transformed and preserved in *E.coli* strain Stbl3. Human DTL and PDCD4 cDNAs were sub-cloned using I5 polymerase (Tsingke, Qingdao). Primer and shRNA sequences were listed in Additional file 9: Table S1.

### Mass spectrometry and protein identification

293 T cells with stable Flag-DTL expression were collected using a scraper and lysed in weak lysis buffer. The cell lysate was incubated with Flag magnetic affinity resin bought from Sigma-Aldrich. SDS-PAGE was performed and gel was stained using Coomassie brilliant blue R250. Protein bands were excised from the gel and then analyzed on LC-MS. Data analysis and protein identification were done by searching against the NCBI protein database.

### Establishment of stable cell lines

Constructed overexpression and silencing plasmids were transfected into 293 T cells, together with package plasmids psPAX and PMD2.G at the ration of 4:3:1 using Transfection Reagent Lipofectamine 2000 (Invitrogen). The supernatants were collected and filtered through 0.4 μm filter membrane after 72 h. Cells were plated in 6-well plates with 1 × 10^6^ cells per well and transfected by collected lentivirus particles. The transfected cells were selected by puromycin for 2 weeks. Establishment of stable cell lines was verified by Western blot.

### Antibodies and reagents

The antibodies used in our experiments are listed in Additional file 10: Table S2. CHX, MG132 were purchased from Calbiochem, protein G/A from Millipore, protease inhibitor cocktail from Roche, TRIzol and Lipofectamine 2000 from Invitrogen and protein lysis buffer RIPA from Beyotime Biotechnology. Whole cell lysates for immunoblots and immunoprecipitation were prepared using RIPA lysis buffer supplemented with protease inhibitors. mRNA was isolated using TRIzol reagent (Invitrogen) according to the manufacturer’s instructions. The reverse-transcription reaction was performed using Revert Aid First Strand cDNA Synthesis Kit (Thermo). Quantitative real-time PCR (qRT-PCR) was performed using SYBR Green PCR Master Mix (CWBIO) and ABI PRISM 7900HT Real-time PCR Detection System (Eppendorf). Primers used for qRT-PCR were listed in Additional file 11: Table S3.

### Bioinformatics analysis

DTL three-dimension structure was predicted by I-TASSER using ab initio protein structure prediction technique [30–32]. PDCD4 three-dimension structure was downloaded from Protein Data Bank (http://www.rcsb.org/) using PDB ID 3EIJ [33]. Protein-protein interaction prediction was performed by ZDOCK server [34]. We used GEO datasets GSE3744, GSE10780, GSE19804 and GSE19188 to confirm DTL expression in breast and lung cancers. Of the two affymetrix ID identified, 218585_s_at was selected as the best probe for DTL. Survival curves were drawn by Kaplan Meier plotter, which was a combination of gene expression and patient survival information from GEO (Affymetrix microarrays only), EGA and TCGA database [35].

### Immunohistochemistry and scoring

Samples of breast cancer, NSNLC tissues and corresponding adjacent non-cancerous tissues were obtained from patients undergoing surgical excision of tumors in Qilu Hospital of Shandong University (Jinan, China). The samples used for paraffin sections were fixed with 10% formalin, and the samples for protein and mRNA extractions were frozen in liquid nitrogen immediately after dissection. Research protocols were approved by the Hospital Ethics Committee of Shandong University and written informed consent was obtained from patients based on the Declaration of Helsinki.

### Cell proliferation, migration and invasion assays

MTT and colony formation assays were performed to assess cell proliferation as described [36]. Wound-healing, transwell and matrigel assays were used to examine cell motility as described [37]. To avoid the affection of cell proliferation on cell motility, cells for transwell and matrigel assays were cultured in a low-serum concentration (0.2%), cells for wound healing assay were cultured without serum.

### Nude mice xenograft and transplanted models

Nude mice (6 weeks old) were purchased from Beijing Huafukang Bioscience Co. INC and maintained in micro-isolator cages in SPF laboratory animal room. All animals were used in accordance with institute guidelines and the experiments were approved by the Use Committee for Animal Care of the institute. For subcutaneous inoculation, cells re-suspended in PBS at a concentration of 2 × 10^7^ cells/mL were injected into 8-week old nude mice. The tumors were measured every 3 days after appearance and the tumor volumes were calculated by the formula (length×width)^2^/2. For metastatic assay, cells with overexpression or knockdown of DTL and corresponding controls were re-suspended in PBS at a concentration of 1 × 10^7^ cells/mL. Cell suspension (0.1 mL) was injected into tail veins of nude mice. The nude mice were sacrificed by anesthesia with chloral hydrate.

### Statistical analysis

Results are expressed as mean ± SD from at least three independent experiments. SPSS17.0 statistical software package (SPSS Inc.) was used for statistical analysis. Statistical differences between groups were assessed using the Student *t* test. Statistics of IHC results were calculated using IOD measurement by Image Pro Plus. Association between DTL and PDCD4 expression in breast and lung cancer tissues was evaluated by the Spearman rank correlation test. *P* < 0.05 was considered statistically significant.

## Results

### DTL combines directly with PDCD4

**Fig. 1:**
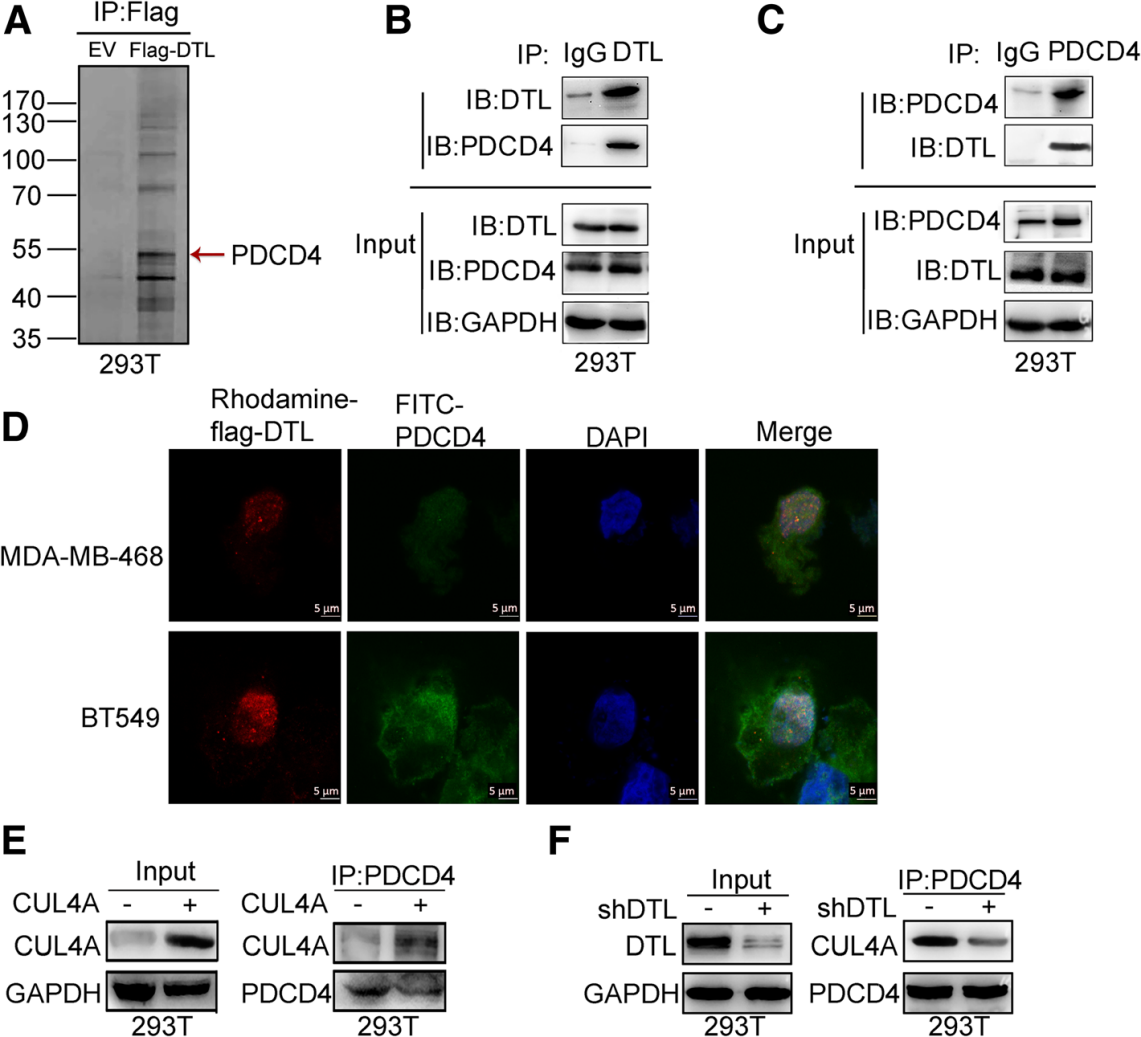
DTL bound with PDCD4. **a** 293 T cells were transfected with Flag-DTL plasmid and the cell lysates were co-immunoprecipitated by Flag magnetic affinity resin. Several differential bands were shown in Coomassie staining SDS-PAGE gels. The band indicated by array showed protein at the position about 55KDa, which was proved to be PDCD4 in the mass spectrometry experiment. **b** 293 T cell lysates were prepared by weak RIPA lysis and co-immunoprecipitated by DTL antibody with IgG as negative control. The result showed that PDCD4 interacts with DTL. **c** Co-immunoprecipitation assay using PDCD4 as bait protein demonstrated the interaction between PDCD4 and DTL. **d** Sub-localizations of DTL and PDCD4 were analyzed using immunofluorescence. Laser Confocal Microscopy photos showed that DTL and PDCD4 were co-localization both in the nuclei and cellular matrix. The scale bars represent 5 μm. **e** CUL4A constructed in PLVX-AcGFP-N1 vector was transient transfected into 293 T cells and immunoprecipitated by PDCD4 antibody. **f** shDTL constructed in PLKO.1-puro was transient transfected into 293 T cells. Interaction between PDCD4 and CUL4A was detected

To investigate how DTL exerting its functions in cancer cells, proteins interacted with DTL were analyzed using immunoaffinity chromatography. 293 T cells were transfected with Flag-DTL plasmid and the cell lysates were co-immunoprecipitated by Flag magnetic affinity resin and followed by SDS-PAGE. After Coomassie blue staining, the bands were excised from gel and detected by matrix assisted laser desorption ionization time of flight mass spectrometry. Tumor suppressor PDCD4 was identified at the position of about 55 kDa (Fig. 1a). Co-immunoprecipitation (Co-IP) assay was carried out to verify the interaction between DTL and PDCD4. Endogenous DTL was immunoprecipitated from 293 T cell lysates by PDCD4 antibody. The interaction was further confirmed by immunoprecipitation of PDCD4 with DTL antibody (Fig. 1b and c). The result was further confirmed in lung cancer cell H1650 (Additional file 1: Figure S1). Sub-localizations of DTL and PDCD4 were analyzed in breast cancer cell lines MDA-MB-468 and BT549 using Laser Confocal Microscopy. Immunofluorescence staining showed that DTL and PDCD4 were co-localization both in the nucleus and cellular matrix (Fig. 1d), providing spatial foundation for their binding. As DTL is one of the DCAFs, we then detected whether CUL4A involved in the binding between DTL and PDCD4. Co-IP demonstrated that CUL4A could interact with PDCD4 (Fig. 1e), and knockdown of DTL weakened the interaction of CUL4A and PDCD4 (Fig. 1f). The results demonstrated that CRL4A E3 ligase combined with PDCD4 through DTL.

**Fig. 2:**
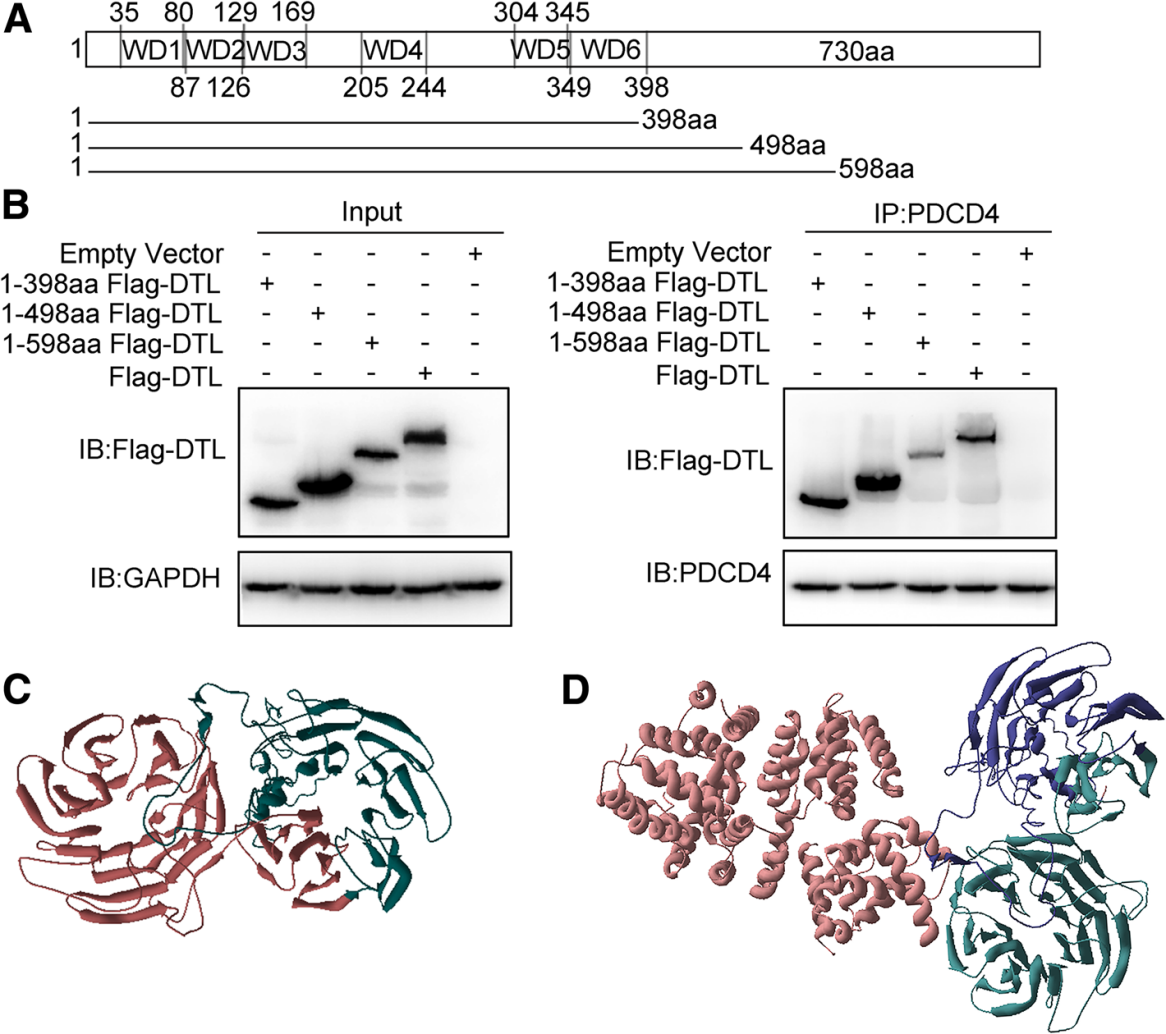
WD40 domains of DTL played important roles in interaction with PDCD4. **a** Schematic diagram of DTL primary structure and truncated plasmid construction. **b** Co-immunoprecipitation assay was performed using truncated DTL fragments. Flag-DTLs were transfected into 293 T cells using Lipofectamine 2000 Transfection reagent. Immunoprecipitation was performed by PDCD4 antibody and immunoblotting by Flag antibody. **c** Three-dimensional structure of DTL was predicted by I-TASSER using ab initio protein structure prediction technique. DTL contained two β propellers, one of them was formed by WD40 domain located in the N-terminal, as shown in red. **d** The interaction between DTL and PDCD4 was shown. PDCD4 was indicated in red, and WD40 domain of DTL in green. The results showed the WD40 domains of DTL interacted with PDCD4

Sequence analysis showed that DTL contains repeated WD40 domains from 35 to 398 amino acids. As indicated in Fig. 2a, truncated fragments of DTL were constructed and transfected into 293 T cells. Cell lysates with DTL fragments were immunoprecipitated with PDCD4 antibody. It turned out that when only WD40 domain of DTL existed, interaction between DTL and PDCD4 was still detectable, suggesting that WD40 domains play an essential role in interaction with PDCD4 (Fig. 2b). Three-dimension structure prediction of DTL showed that it contained two β propellers, one of them were WD40 domain, as indicated in red (Fig. 2c). Interaction prediction results showed in Fig. 2d indicated DTL directly interacted with PDCD4 and WD40 may be involved the interaction.

### DTL degrades PDCD4 through ubiquitin-proteasomal pathway

**Fig. 3:**
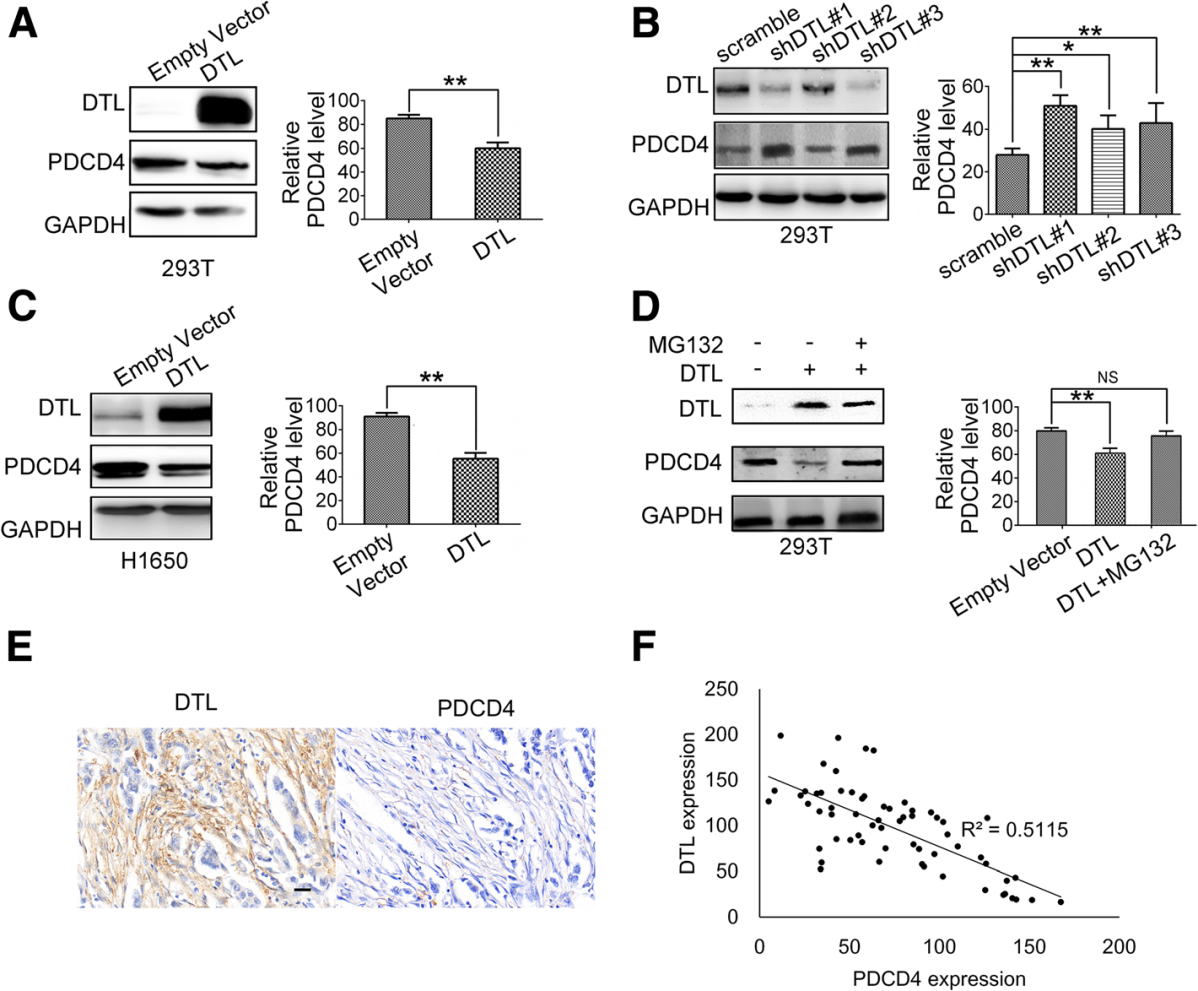
DTL overexpression reduced PDCD4 protein level. **a** DTL cDNA was constructed into PLVX-AcGFP-N1 vector and transfected into 293 T cells. PDCD4 protein level was detected using Western blotting. **b** Three different DTL interference sequences with a scramble sequence were constructed in PLKO.1-puro vector and transfected into 293 T cells. PDCD4 protein level was detected. **c** DTL stable overexpression cell line was established in H1650 and the PDCD4 protein level was detected. **d** 10 mmol/L MG132 was added for 6 h into 293 T cells with DTL overexpression. The PDCD4 protein level was detected by Western blot. **e** Breast cancer tissues were collected and stained by IHC using DTL and PDCD4 antibodies. The scale bar represents 50 μm. **f** Quantitative analysis were performed by Image-pro Plus software and correlation analysis was performed by EXCEL. *, *P* < 0.05, **, *P* < 0.01, NS, no significance based on the Student *t* test. All results are from three or four independent experiments. Error bars, SD

To verify whether PDCD4 was one of the substrates of DTL, the influence of DTL on PDCD4 expression level was analyzed. Detecting of PDCD4 mRNA level showed that DTL did not influence PDCD4 expression in transcriptional level (Additional file 2: Figure S2). Overexpression of DTL in 293 T cells caused the decline and silencing DTL led to the accumulation of PDCD4 in protein level (Fig. 3a and b). The same result was confirmed in cancer cell line H1650 (Fig. 3c). In DTL overexpression cells, MG132 inhibited the PDCD4 degradation as shown in Fig. 3d. To further investigate the correlation of DTL and PDCD4 expression, breast cancer tissues were collected and IHC staining were performed on paraffin sections. The IHC results showed that PDCD4 expression was negatively correlated with DTL (Fig. 3e and f).

**Fig. 4:**
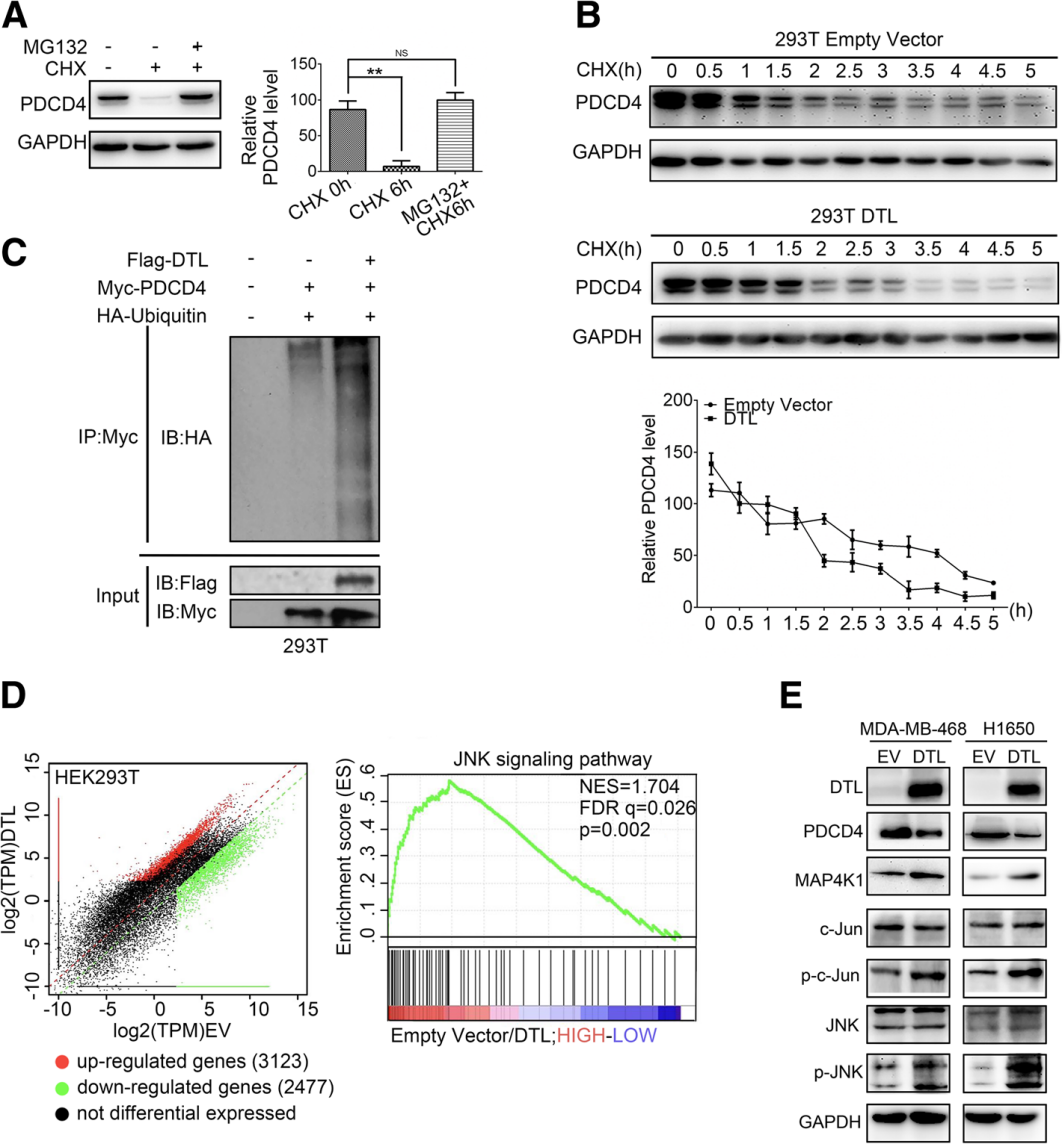
DTL degraded PDCD4 and activated JNK pathway. **a** 293 T cells treated with 50 mg/mL cycloheximide alone or plus 10 mmol/L MG132 for 6 h. PDCD4 expression was detected. **b** 293 T cells were transient transfected by DTL or empty vector and treated with 50 mg/mL cycloheximide (CHX) for indicated times. The diagram showed quantitative analysis of PDCD4 levels. **c** HA-ubiquitin and Myc-PDCD4 were co-transfected into 293 T cells with or without DTL. Immunoprecipitation with anti-Myc antibody followed by Western blot using HA antibodies showed DTL promotes PDCD4 ubiquitination level. **d** RNA sequencing was performed in 293 T cells with empty vector and DTL respectively. Pathway enrichment analysis of differential expression genes revealed that JNK pathway was the most enriched. **e** Western blot was used to confirm the results obtained by RNA sequencing in cancer cells. *, *P* < 0.05, **, *P* < 0.01 based on the Student *t* test. All results are from three or four independent experiments. Error bars, SD

To investigate the mechanisms of PDCD4 degradation, protein synthesis inhibitor cycloheximide (CHX) and proteasome inhibitor MG132 were added into the cultured cells. In the presence of CHX, PDCD4 protein level was dramatically decreased and MG132 treatment recovered PDCD4 protein level (Fig. 4a). This phenomenon suggested that ubiquitin system played important role in PDCD4 degradation. Further, under the conditions that protein synthesis was inhibited, exogenous DTL dramatically reduced PDCD4 level within 3 h, indicating that DTL accelerated the degradation rate of PDCD4 (Fig. 4b). To verify whether DTL affects the level of PDCD4 ubiquitination, ubiquitin and PDCD4 were co-transfected into 293 T cells. PDCD4 was efficiently ubiquitylated when DTL was overexpressed, indicating that DTL promoted ubiquitin modification level of PDCD4 (Fig. 4c). Our results proved that DTL combined with PDCD4 and promoted the ubiquitin-proteasomal degradation of PDCD4.

As phosphorylation of PDCD4 at Ser67 has been proved to play important roles in SCF ligase mediated degradation, we then detected whether phosphorylation of PDCD4 at Ser67 influenced the binding between PDCD4 and DTL. A mutant form of PDCD4 was constructed by substituting Ser67 to Ala67. Alanine was structurally similar to serine, which could reduce phosphorylation level without affecting the structure of PDCD4. As shown in Additional file 3: Figure S3, the interaction between PDCD4(S67A) and DTL was not detected, indicated that the phosphorylation of Ser67 played an important role in its binding with DTL.

To further explore potential functions of DTL in cancers, gene expression profiling on 293 T-DTL and its control cells was employed. Among the 5600 different expression genes identified, several pathways were enriched in GSEA pathway analysis, including cancer related JNK pathway (Fig. 4d). According to the previous study that silencing PDCD4 induced MAP4K1 expression thus gave rise to JNK activation [38], the impact of DTL on JNK pathway was further explored. As shown in Fig. 4e, DTL overexpression up-regulated the phosphorylation levels of c-JUN and JNK (marked as p-c-JUN and p-JNK). Our results demonstrated that DTL ubiquitination degraded PDCD4 and activated JNK pathway.

### DTL is commonly up-regulated in cancer tissues and related to poor outcomes

**Fig. 5:**
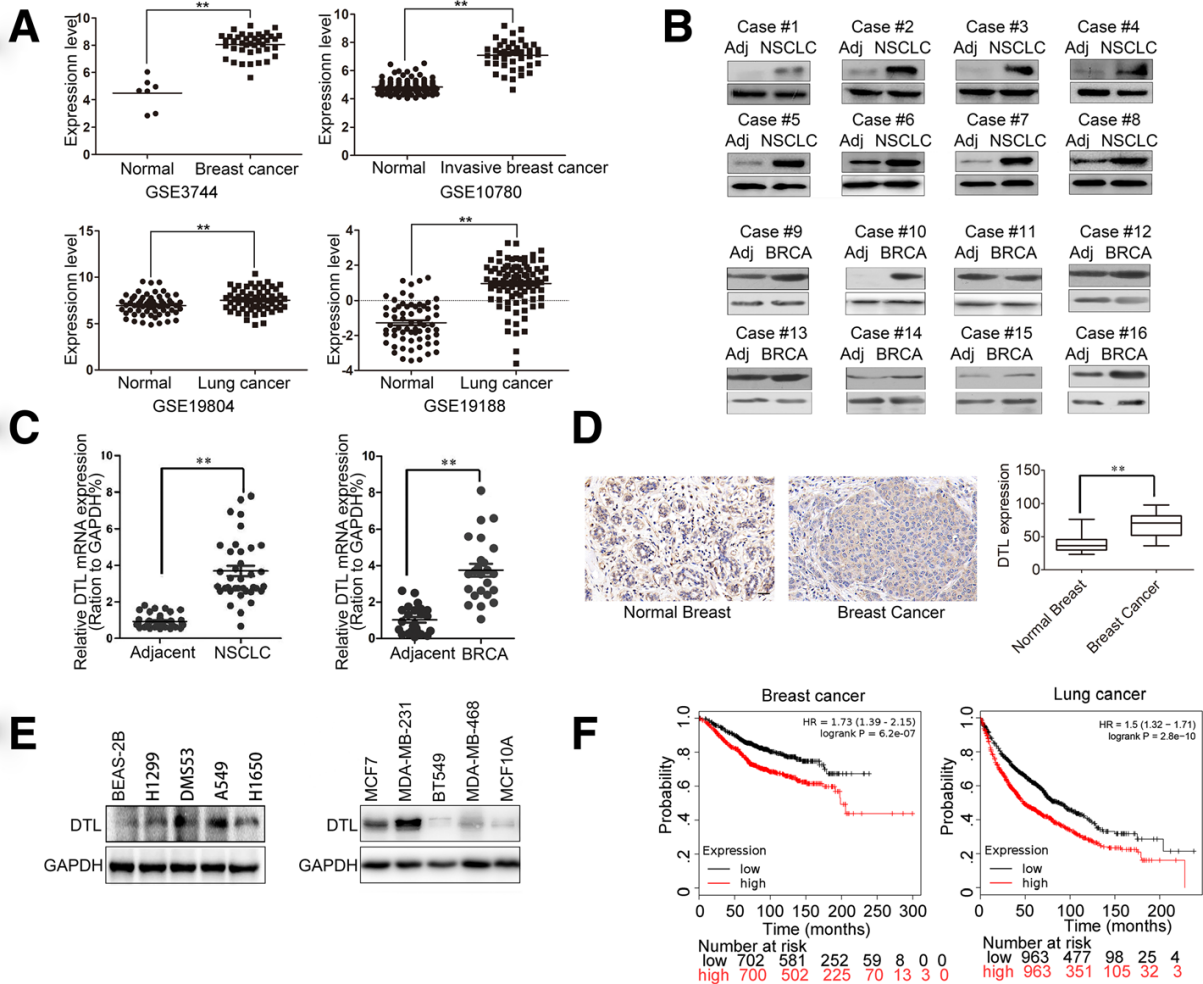
DTL overexpression in cancer tissues and cells was related to patient prognosis. **a** GEO database analysis of DTL expression in breast and lung cancers. **b** Proteins of eight pairs of lung and breast cancer tissues were extracted. Western blot analysis showed DTL expression levels in cancer and adjacent tissues. NSCLC means non-small cell lung cancer and BRCA means breast cancer. **c** DTL mRNA levels of 37 pairs of lung cancer and 24 pairs of breast cancer with their adjacent normal tissues were detected by real-time quantitative PCR. **d** IHC staining of human breast tissues and breast cancer tissues for DTL to show their expression differences. Statistic results were shown in right diagram using IOD analysis in Image Pro Plus software. The scale bar represents 50 μm. **e** Western blot to assess the DTL protein expression in lung and breast cancer cell lines. **f** Survival curves of breast and lung cancer patients with alteration DTL levels were calculated in Kaplan-Meier method using this KM plotter. The patient numbers were shown below each diagram. *, *P* < 0.05, **, *P* < 0.01 based on the Student *t* test. All results are from three or four independent experiments. Error bars, SD

To get an overall profile of DTL expression in cancers, we analyzed DTL gene expression levels in 37 types of cancers (http://firebrowse.org/) [39]. Almost all cancer tissues analyzed showed higher DTL level than normal controls (Additional file 4: Figure S4). GEO database analysis also confirmed that expression level of DTL in breast and lung cancer tissues were higher than that in normal tissues (Fig. 5a). Furthermore, using our collected cancer tissues with adjacent normal tissues, mRNA and protein levels of DTL were analyzed. Eight pairs of lung and breast cancer tissues and their corresponding adjacent tissues were analyzed by Western blot (Fig. 5b). 37 pairs of lung cancers and 24 pairs of breast cancers with their relative adjacent normal tissues were analyzed by qRT-PCR (Additional file 5: Figure S5, Fig. 5c). 92 breast cancer tissue samples (11 of them with paired adjacent non-tumorous tissues) were analyzed by IHC (Fig. 5d). Consistent with the above results, DTL was significantly highly expressed in breast and lung cancer tissues. DTL expression in breast and lung cancer cell lines also showed significantly higher levels than that in the normal cells (Fig. 5e). To verify the effect of DTL on prognosis of cancer patients, survival curves were charted by Kaplan-Meier plotter. As shown in Fig. 5f, high expression of DTL shortened the survival time not only of the breast cancer, but also the lung cancer patients. Above all, DTL was overexpressed in cancer tissues and related to the poor outcomes of cancer patients.

### DTL enhances proliferation, migration and invasion of cancer cells in vitro and in vivo

**Fig. 6:**
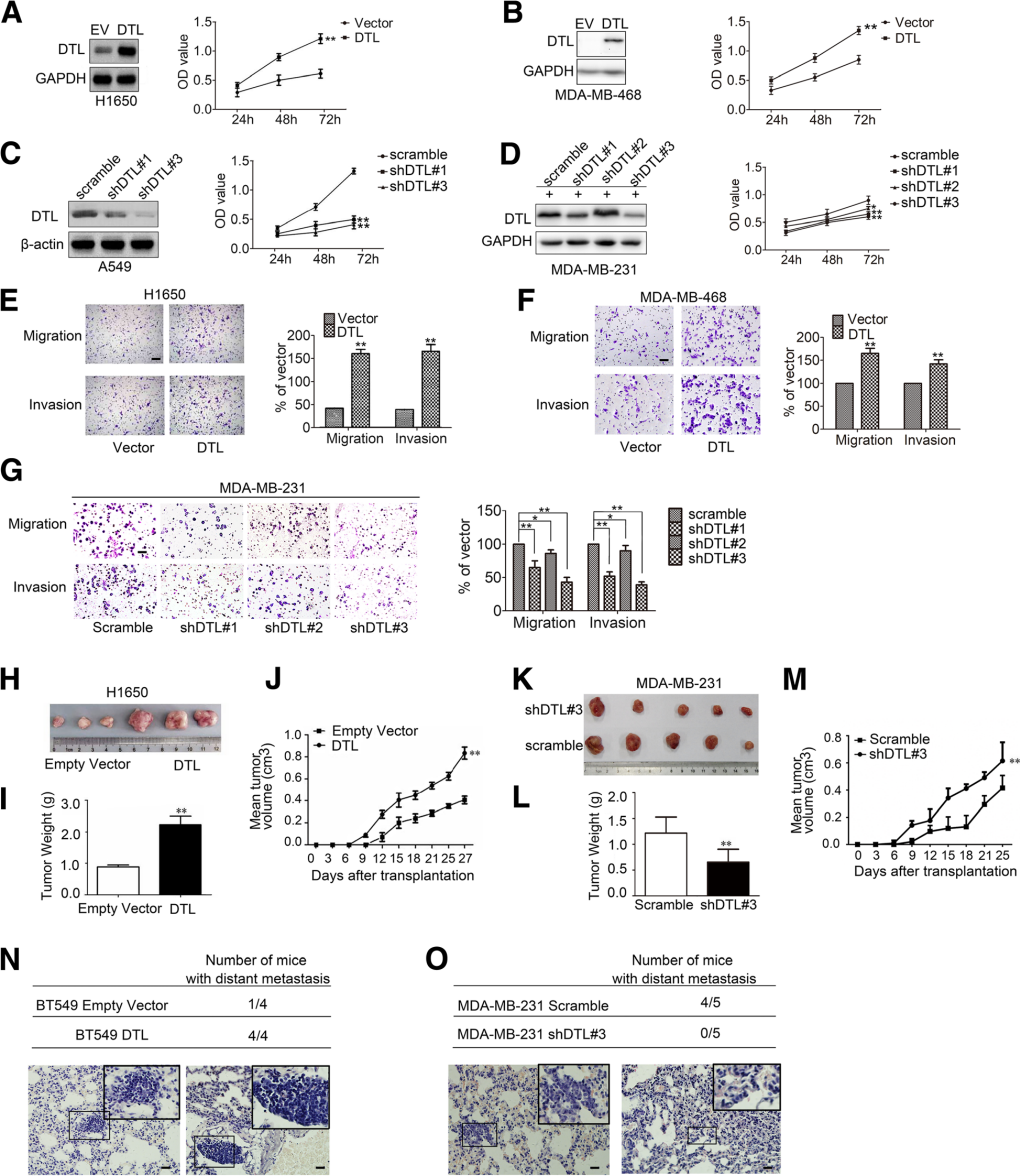
DTL promoted proliferation and motilities of lung and breast cancer cells. **a**-**b** Lung cancer cell line H1650 (**a**) and breast cancer cell line MDA-MB-468 (**b**) were used to establish DTL overexpression cell lines. Cell proliferation was examined by MTT. **c**-**d** Lung cancer cell line A549 (**c**) and breast cancer cell line MDA-MB-231 (**d**) were used to establish DTL silencing cell lines. Cell proliferation was examined by MTT. **e**-**f** Transwell and Matrigel assays were carried out in DTL overexpression cell lines H1650 (**e**) and MDA-MB-468 (**f**). **g** Transwell and Matrigel assays were carried out in DTL silencing cell line MDA-MB-231. **h** Nude mice were hypodermically injected with DTL overexpression cell line H1650. The nude mice were sacrificed by anesthesia with chloral hydrate and the transplanted cancer tissues were dissected out. **i** Tumor weights were measured after dissected. **j** The tumor volumes were measured during their growth process using ruler, calculating by the formula (length×width)^2^/2. **k** Nude mice were hypodermiclly injected with silencing cell line MDA-MB-231. The nude mice were sacrificed by anesthesia with chloral hydrate and the transplanted cancer tissues were dissected out. **l** Tumor weights were measured after dissected. **m** The tumor volumes were measured during their growth process and calculating by the formula (length×width)^2^/2. **n**-**o** Total numbers of nude mice with distant metastasis at 40 to 50 days after tail vein injection of DTL overexpression cell line BT549 and DTL silence cell line MDA-MB-231. Lung tissues were dissected out and made into paraffin sections after the nude mice were sacrificed. Distant metastasis were observed after hematoxylin and eosin (HE) staining. The lower panel showed the representative HE staining. The scale bars represent 50 μm. *, *P* < 0.05, **, *P* < 0.01 based on the Student *t* test. All results are from three or four independent experiments. Error bars, SD

The influences of DTL on the vitality and mobility of cancer cells were then investigated. Cancer cell lines H1650, MDA-MB-468 and BT549 were used to establish DTL stable overexpression cell lines by lentivirus. DTL silencing cancer cell lines were established using lung cancer cell A549 and breast cancer cell MDA-MB-231 respectively by lentivirus. MDA-MB-231 cell line with DTL silence was constructed by doxycycline inducible plasmids since DTL knockdown in this cell line led to poor growth status. The optimum induction conditions were examined as Additional file 6: Figure S6. The results of MTT and colony formation assays showed that DTL significantly increased the growth rates of cancer cells (Fig. 6a and b, Additional file 7: Figure S7A-C). In turn, silencing DTL slowed down the cancer cell growth rates and weakened the colony formation abilities of cancer cells (Fig. 6c and d, Additional file 7: Figure S7D and E). Effects of DTL on migration and invasion of cancer cells were detected. Overexpression of DTL increased the numbers of cell migrating through the membrane to the bottom of the aperture, and transferred through the matrigel layer more easily than that of empty vector (Fig. 6e and f). In contrast, Silencing DTL reduced the cell numbers that passed through the aperture with or without matrigel (Fig. 6g). Same results were obtained in BT549 and A549 cell lines (Additional file 7: Figure S7F and G).

Moreover, to detect the functions of DTL for cancer cells in vivo, H1650 and MDA-MB-231 cells with DTL overexpression and knocking down were hypodermically injected into nude mice respectively. The results indicated that DTL overexpression promoted tumor growth (Fig. 6h-j) and DTL knockdown inhibited tumor growth in terms of tumor weight and volumes (Fig. 6k-m). To detect the cancer cell mobility in vivo, established breast cancer cell lines with overexpression and silencing DTL were injected into nude mice through the tail vein respectively. Paraffin sections were stained with Hematoxylin-Eosin to identify the metastatic foci. Overexpression DTL significantly increased the number of mice with distant metastasis, while silencing DTL reduced the distant metastasis (Fig. 6n and o). Immunohistochemical analysis showed the expression of PDCD4 levels in mice xenograft tumors were negatively correlated with DTL expression (Additional file 7: Figure S7H).

### PDCD4 mediates DTL functions in cancer cells

**Fig. 7:**
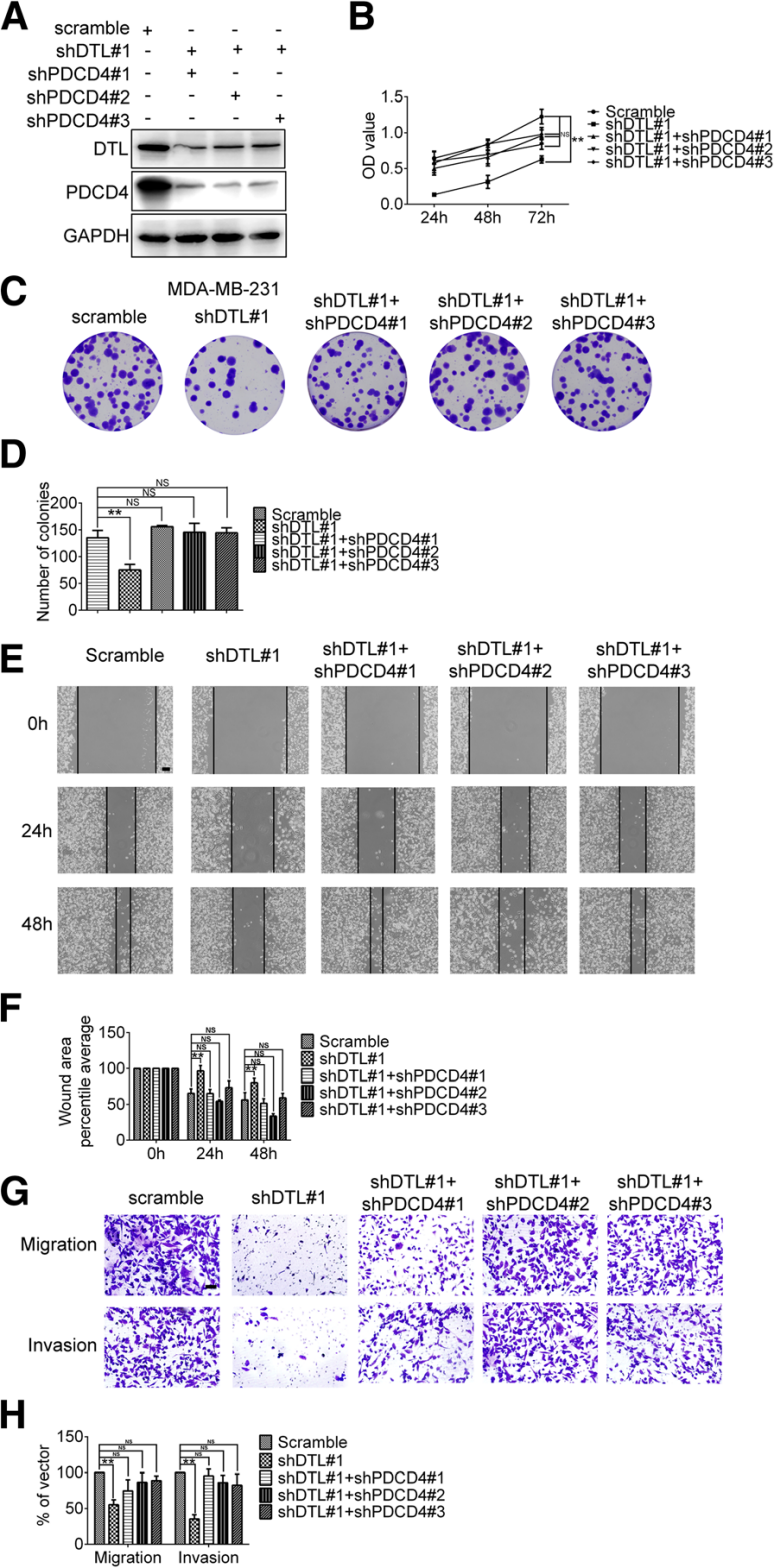
PDCD4 mediated DTL functions in cancer cells. **a** Three different PDCD4 interference sequences and a scramble sequence were constructed in PLKO.1-puro vector and packaged into lentivirus to infect MDA-MB-231 cells. Knockdown efficiency was detected by Western blot. **b** MTT assay results showed that knockdown of PDCD4 rescued the proliferation ability insufficient in DTL silencing cells. **c** Colony formation assay was performed in MDA-MB-231 cells. **d** Statistical analysis results of colony formation were as shown. **e** Representative transwell and matrigel assays performed in MDA-MB-231 cells were shown. **f** Statistical analysis of transwell and matrigel assays. **g** Wound healing assay was performed in the MDA-MB-231 cells with silencing both DTL and PDCD4. Uncovered areas in the wound healing assay were quantified as a percentage of the original wound area. **h** Statistical analysis of wound healing assays. The scale bars represent 50 μm. *, *P* < 0.05, **, *P* < 0.01, NS, no significance based on the Student *t* test. All results are from three or four independent experiments. Error bars, SD

To detect whether DTL influence cancer cell proliferation and motility abilities via PDCD4, rescued functional experiments were performed. Breast cancer cell line MDA-MB-231 with DTL silence was used to establish PDCD4 knockdown cell lines. The interference efficiency was detected by Western blotting as shown in Fig. 7a. MTT and colony formation assays revealed that the PDCD4 down-expression recovered cell proliferation ability impaired by DTL silencing to some extent (Fig. 7b-d). Wound healing and transwell assays were used to detect the migration ability of double silencing cells. The results showed that the migration ability has been rescued by PDCD4 knocking down (Fig. 7e-h). Results in Fig. 7g and h also showed that in PDCD4 knockdown cells, cell numbers invaded through the matrigel layer to the bottom of the inserts were recovered to the cell number with empty vectors. These results demonstrated that the defection caused by DTL silencing was offset by down regulation of PDCD4.

As described above, we proved that DTL overexpression induced the down-regulation of PDCD4 level and silencing PDCD4 is reported to induce the activation of JNK pathway [38], we then used JNK inhibitor JNK-IN-8 to detect whether JNK pathway participated in the regulation of cell motility and proliferation abilities in DTL overexpression cells. 5 μM JNK-IN-8 was added into DTL overexpression cells for 3 h (Additional file 8: Figure S8A). MTT and colony formation assays showed that JNK inhibitor reduced the proliferation ability of MCF7 and BT549 cells (Additional file 8: Figure S8B-D). Also, as shown in Additional file 8: Figure S8E and F, cells with JNK inhibitor exhibited lower capability of migration and invasion.

## Discussion

DTL is identified as one of the DCAFs of CUL4A and plays important roles in cell cycle and DNA repair. In our study, DTL overexpression increased PDCD4 ubiquitination level and decreased PDCD4 protein level. Overexpression of DTL accelerated tumor cell growth and increased invasive ability of cancer cells. Moreover, silencing PDCD4 rescued cancer cell growth and mobility deficiencies caused by DTL knockdown. To our knowledge, this is the first report to show that DTL played roles in degrading tumor suppressor PDCD4.

Previous studies showed that CUL4A played a functional role in metastasis in cancer cells by inducing proliferation, EMT, migration, and invasion [37]. DCAFs including DTL interact with DDB1 thus to CUL4A to help receive substrates and exert variable functions [6]. Several substrates of DTL have been identified such as CDT1, P21, PR-Set7/Set8. As essential for proper genome replication [40, 41], CDT1 is considered to play an important role in mediating DTL induced cancer cell proliferation. It is reported DTL depletion in cancer cells caused apoptotic death of cancer cells associated with rereplication due to the loss of CDT1 degradation, but not in non-transformed cells [42]. Here we reported that PDCD4 might be another mediator in DTL regulating cancer cell proliferation.

Our studies revealed that DTL interacted with PDCD4 directly. Sequence analysis showed that DTL has seven WD40 domains in the N-terminal with two WDXR modules. Detailed analysis of protein interaction revealed that PDCD4 bind to WD40 domains of DTL directly. In addition, using ab initio protein structure prediction, we found that DTL has two β-propeller, one of them was formed by WD40 domain in the N-terminal, which has been proved to play important roles in binding with PDCD4. Further studies would be needed for the structural analysis of DTL and DDB1-CUL4A complex.

Programmed cell death 4 (PDCD4) was generally known as tumor repressor inhibiting translation initiation by displacing eIF4G and RNA from eIF4A [43]. PDCD4 was down-regulated or loss in many cancers and related to tumor progression and poor outcomes, such as colon cancer, ovarian cancer, glioblastoma and lung cancer [25–28]. Previous studies showed that PDCD4 was degraded by two ways, miR-21 and SCF^βTRCP^ mediated degradation [23, 44, 45]. Our study provided evidences that overexpression DTL decreased PDCD4 expression level and promote the ubiquitin level of PDCD4 in cells. In addition, previous studies demonstrated that silencing PDCD4 up-regulates MAP4K1 expression thus to active AP-1 dependent transcription [38, 46]. In this study, we also found that DTL induced MAP4K1 expression and enhanced phosphorylation levels of JNK and c-Jun proteins. These findings suggested that DTL degraded PDCD4 to active JNK pathway.

## Conclusions

In conclusion, our study firstly reported the interaction between DTL and PDCD4. DTL overexpression elevated PDCD4 ubiquitin level and accelerated PDCD4 degradation. Functional assays revealed that DTL increased proliferation, migration and invasive abilities of cancer cells, as well as in vivo tumor genesis abilities. Also, PDCD4 recovered cell growth deficiencies caused by DTL knocking down in proliferation and motilities. Our results suggested DTL promote cancer progression through degrading PDCD4.

## Additional files

Additional file 1: **Figure S1.** DTL interacted with PDCD4 in H1650 cells. (TIF 817 kb)

Additional file 2: **Figure S2.** mRNA levels of PDCD4 in DTL silencing cells. (TIF 916 kb)

Additional file 3: **Figure S3.** HA-DTL, His-PDCD4 and His-PDCD4(S67A) were transfected into 293 T cells as shown. Co-immunoprecipitation assay using His tag antibodies showed that DTL did not binding with S67A mutant PDCD4. (TIF 233 kb)

Additional file 4: **Figure S4.** DTL was commonly over expressed in cancer tissues. The full names of abbreviations were listed below. (TIF 110 kb)

Additional file 5: **Figure S5.** mRNA levels of DTL in lung and breast cancer tissues and normal tissues was shown in histogram. (TIF 1490 kb)

Additional file 6: **Figure S6**. Doxycycline induced DTL knockdown in MDA-MB-231 cells with transfection of Tet-PLKO-puro plasmid. Various time points and concentrations according to references were used as shown. 0.5 μg/mL of doxycycline for 48 h treatment was chosen for further experiments. (TIF 1368 kb)

Additional file 7: **Figure S7.** DTL promoted cancer progress and was negatively correlated with PDCD4. (A) Cell proliferation in vitro was examined by MTT in DTL overexpression cell line BT549. (B-C) Colony formation assay showed DTL overexpression enhanced proliferation ability of H1650 (B) and MDA-MB-468 (C). (D-E) Colony formation assay showed DTL silencing reduced proliferation ability of A549 (D) and MDA-MB-231 (E). (F) Overexpression of DTL in BT549 cells enhanced invasion and migration abilities. (G) Silence of DTL expression in A549 inhibites invasion and migration abilities. (H) Paraffin sections of xenograft tumors were stained with DTL and PDCD4 antibodies. Statistic analysis were shown in right diagram using IOD analysis in Image Pro Plus software and revealed the negative correlation between DTL and PDCD4 expression. *, *P* < 0.05, **, *P* < 0.01 based on the Student *t* test. All results are from three or four independent experiments. Error bars, SD. (TIF 35146 kb)

Additional file 8: **Figure S8.** (A) 5 μM JNK-IN-8 was added into DTL overexpression cells for 3 h. Protein levels of p-c-Jun were detected. (B-D) MTT (B and C) and colony formation (D) assays showed that JNK inhibitor reduced the proliferation ability of MCF7 and BT549 cells. (E-F) MCF7 (E) and BT549 (F) cells with DTL or empty vectors were added 5 μM JNK-IN-8. Transwell and Matrigel assays showed that JNK inhibitor reduced the migration and invasion abilities of cancer cells. Statistical analysis results were shown in the right panel. *, *P* < 0.05, **, *P* < 0.01, NS, no significance based on the Student *t* test. All results are from three or four independent experiments. Error bars, SD. (TIF 7116 kb)

Additional file 9: **Table S1.** The primer sequences used for plasmids construction were listed. (DOC 35 kb)

Additional file 10: **Table S2.** The antibodies used and their corresponding bands were listed. (DOC 33 kb)

Additional file 11: **Table S3.** Primers for Quantitative real-time PCR were listed. (DOC 28 kb)

## Data Availability

The datasets used and/or analyzed during the current study are available from the corresponding author on reasonable request.

## Abbreviations

CHX: Cycloheximide
Co-IP: Co-immunoprecipitation
CUL4A: Cullin4A
DCAF: DDB1 cullin4 associated factor
DTL: Denticleless E3 ubiquitin protein ligase homolog
eIF4A: Eukaryotic initiation factor-4A
eIF4G: Eukaryotic initiation factor-4G
JNK: c-Jun N-terminal kinase
MTT: 3-(4,5- dimethylthiazol-2-yl)-2,5 diphenyltetrazolium bromide
NSCLC: Non small cell lung cancer
PDCD4: Programmed cell death protein 4
SD: Standard deviation
UTR: Untranslated Region
