# Supplementary figures and images for: DTL promotes cancer progression by PDCD4 ubiquitin-dependent degradation
Source: J Exp Clin Cancer Res. 2019 Aug 13;38:350. doi: 10.1186/s13046-019-1358-x (PMC6693180; doi:10.1186/s13046-019-1358-x)

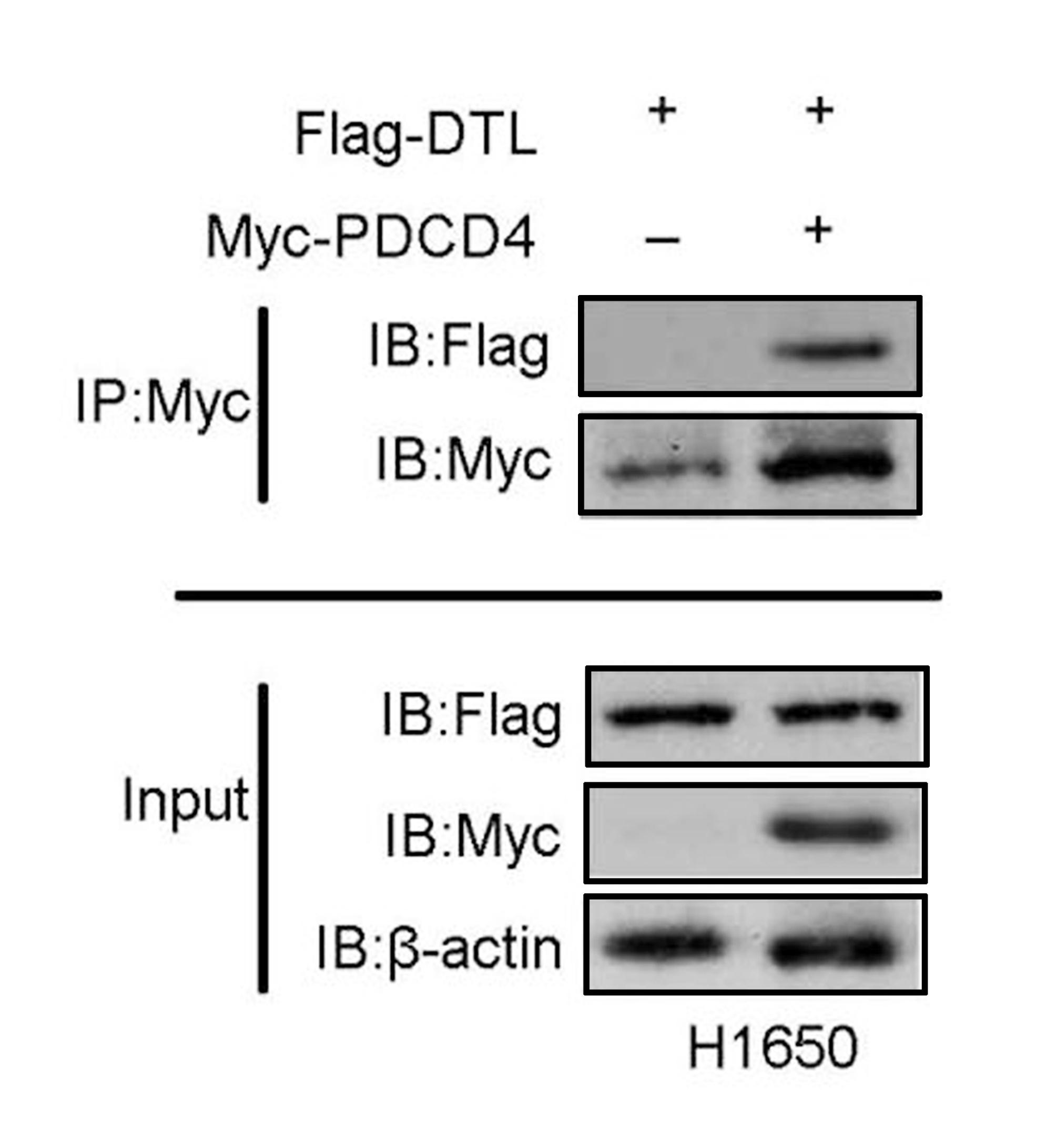

Supplement: Supplementary file 1 — Figure S1. DTL interacted with PDCD4 in H1650 cells. (TIF 817 kb) [file 13046_2019_1358_MOESM1_ESM.tif]

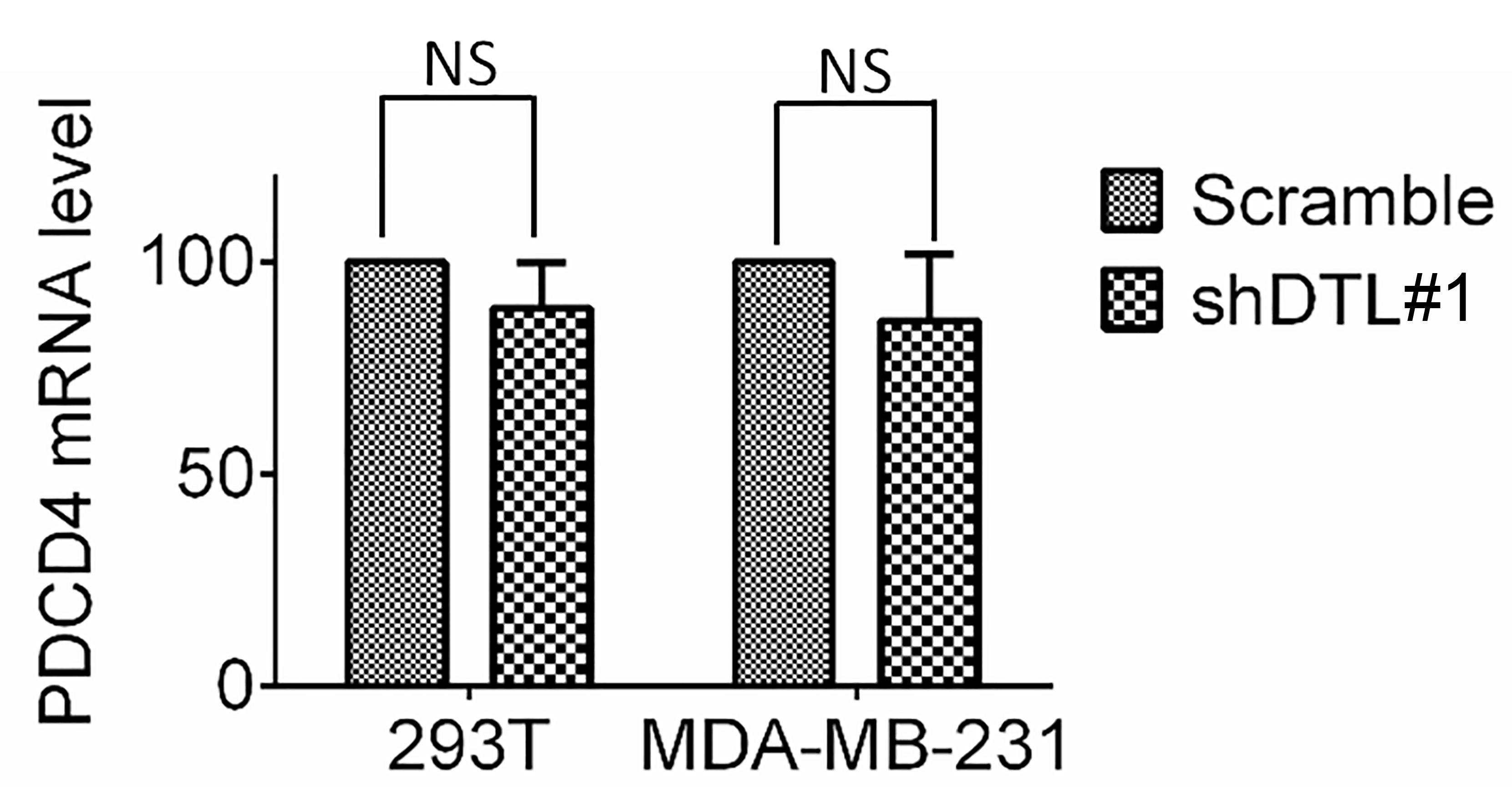

Supplement: Supplementary file 2 — Figure S2. mRNA levels of PDCD4 in DTL silencing cells. (TIF 916 kb) [file 13046_2019_1358_MOESM2_ESM.tif]

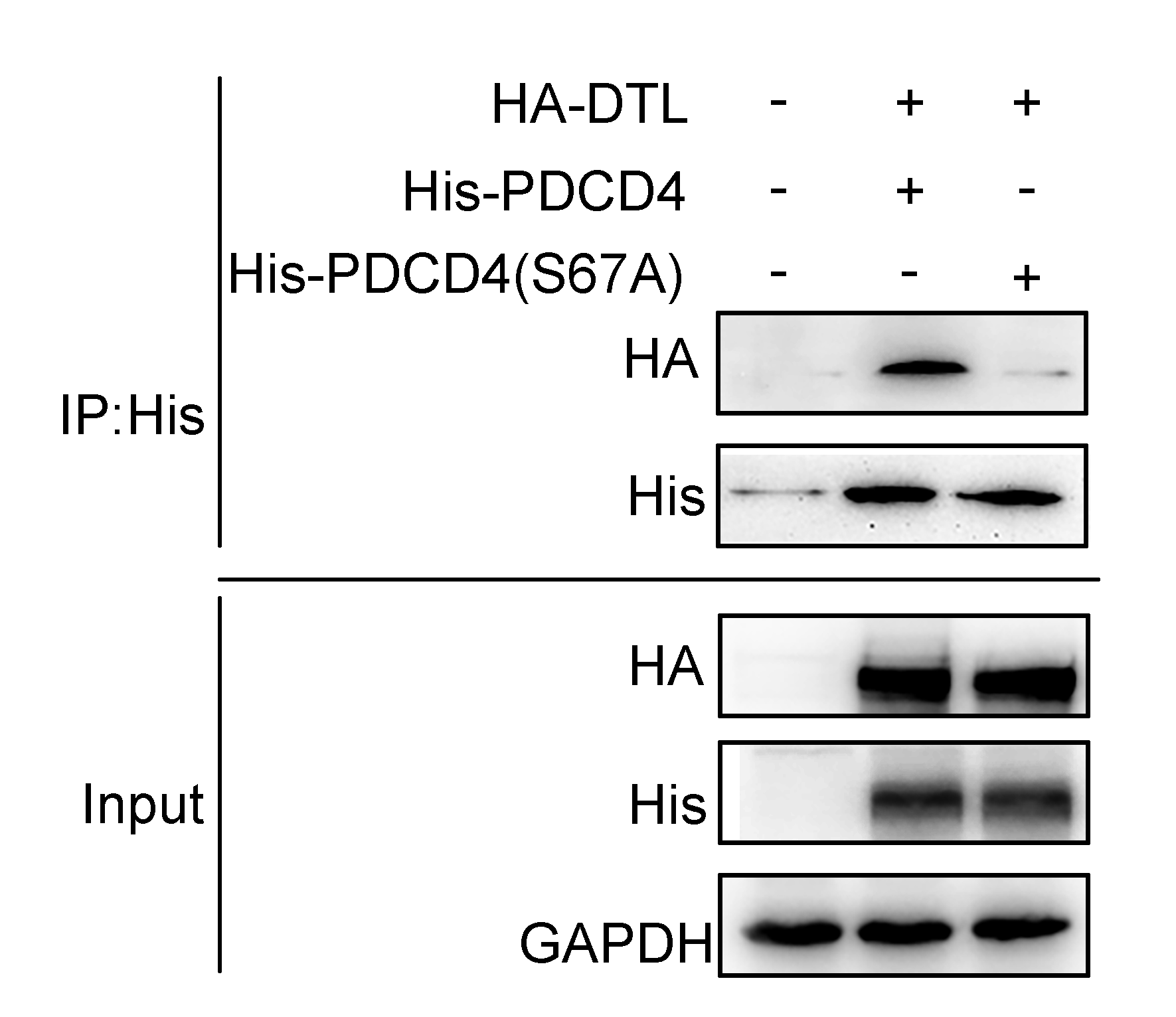

Supplement: Supplementary file 3 — Figure S3. HA-DTL, His-PDCD4 and His-PDCD4(S67A) were transfected into 293 T cells as shown. Co-immunoprecipitation assay using His tag antibodies showed that DTL did not binding with S67A mutant PDCD4. (TIF 233 kb) [file 13046_2019_1358_MOESM3_ESM.tif]

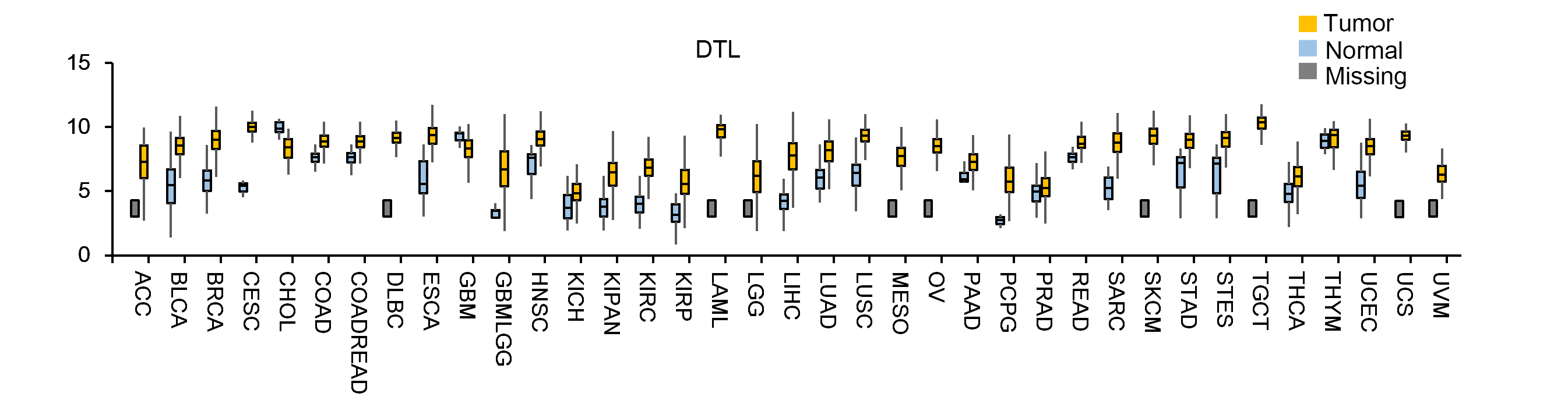

Supplement: Supplementary file 4 — Figure S4. DTL was commonly over expressed in cancer tissues. The full names of abbreviations were listed below. (TIF 110 kb) [file 13046_2019_1358_MOESM4_ESM.tif]

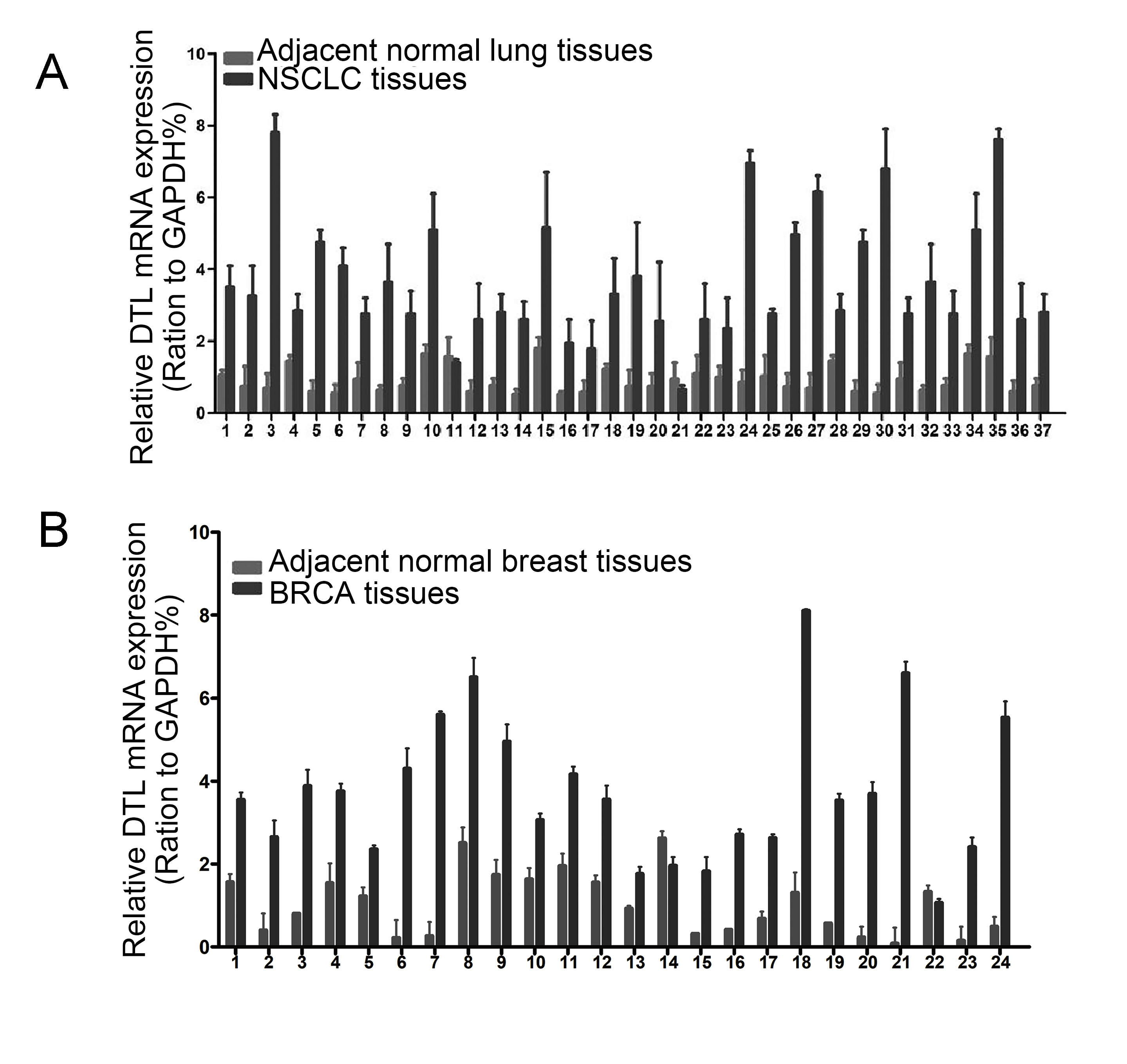

Supplement: Supplementary file 5 — Figure S5. mRNA levels of DTL in lung and breast cancer tissues and normal tissues was shown in histogram. (TIF 1490 kb) [file 13046_2019_1358_MOESM5_ESM.tif]

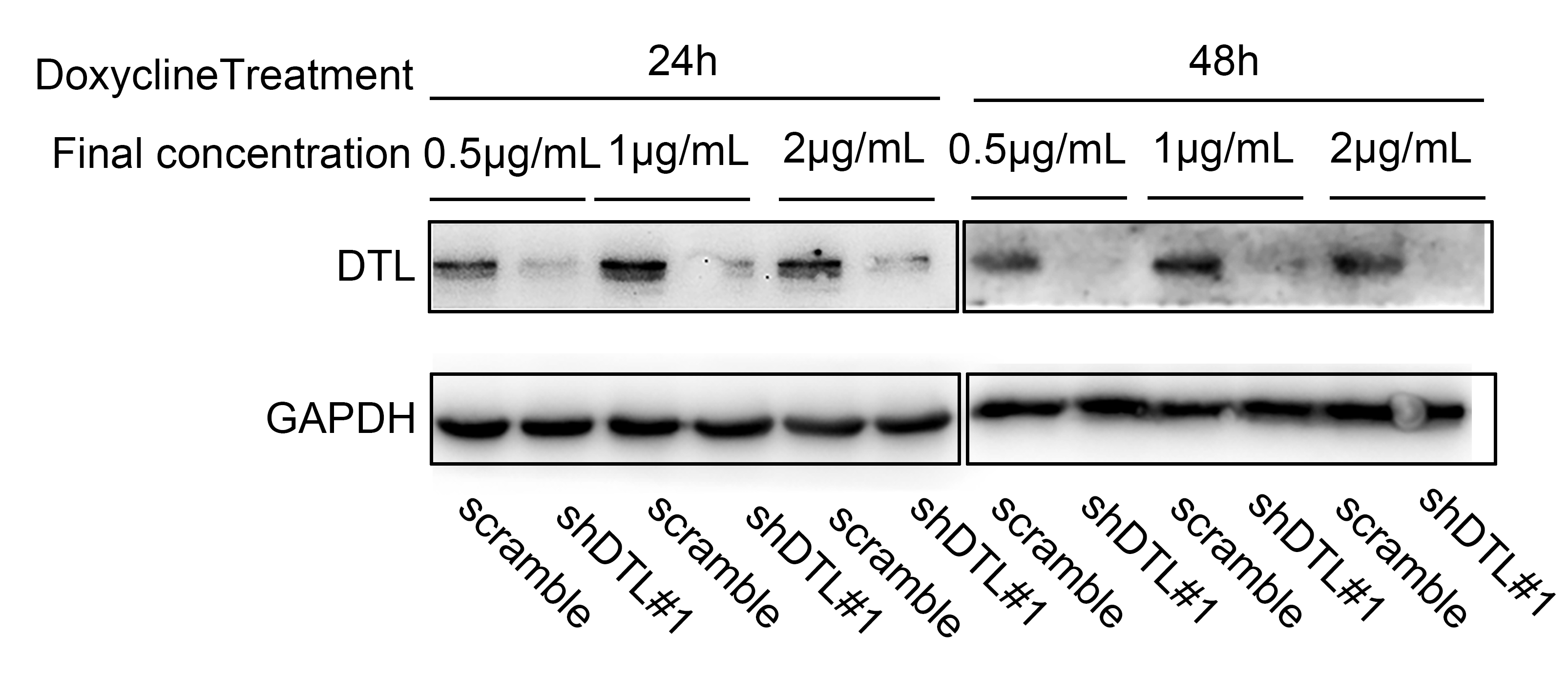

Supplement: Supplementary file 6 — Figure S6. Doxycycline induced DTL knockdown in MDA-MB-231 cells with transfection of Tet-PLKO-puro plasmid. Various time points and concentrations according to references were used as shown. 0.5 μg/mL of doxycycline for 48 h treatment was chosen for further experiments. (TIF 1368 kb) [file 13046_2019_1358_MOESM6_ESM.tif]

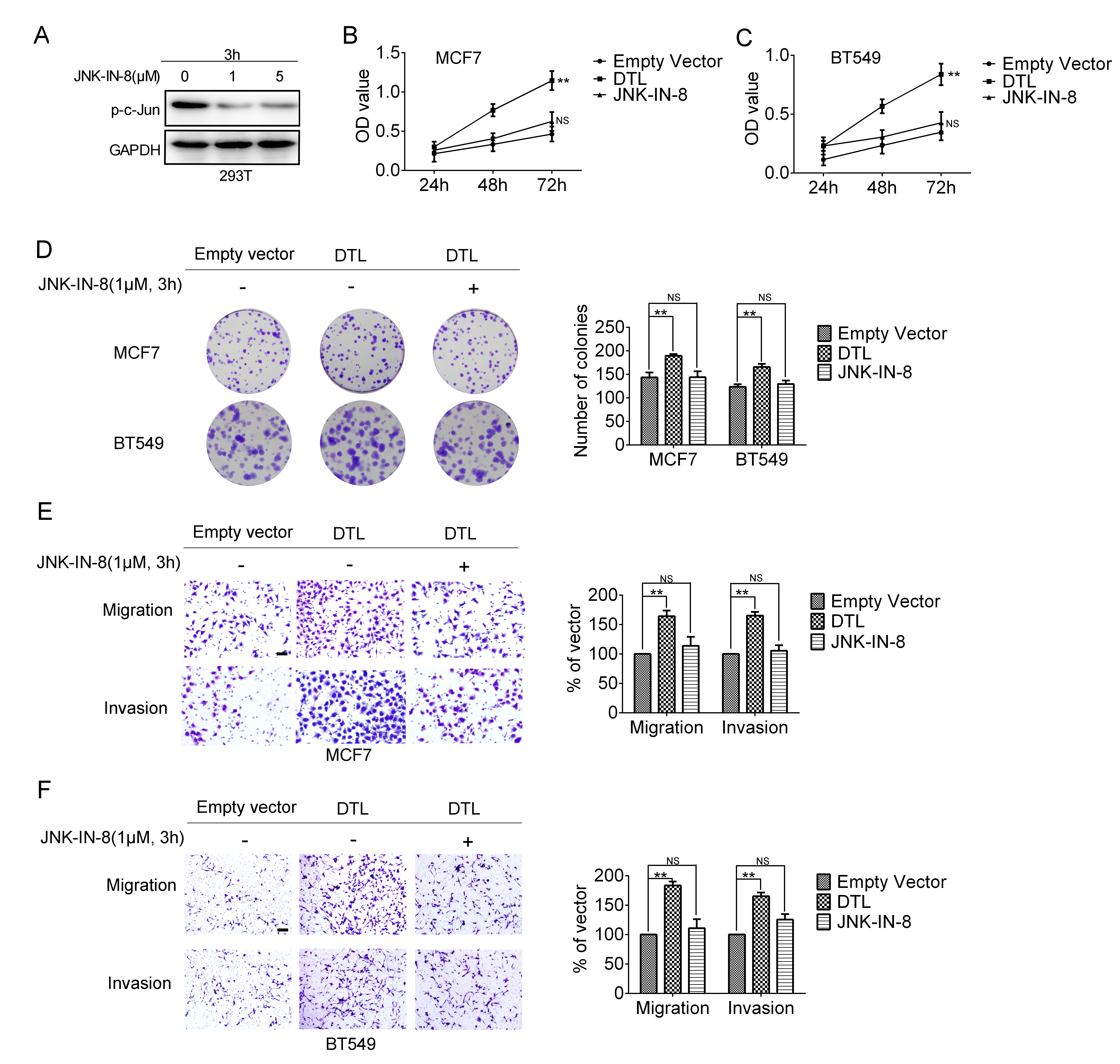

Supplement: Supplementary file 8 — Figure S8. (A) 5 μM JNK-IN-8 was added into DTL overexpression cells for 3 h. Protein levels of p-c-Jun were detected. (B-D) MTT (B and C) and colony formation (D) assays showed that JNK inhibitor reduced the proliferation ability of MCF7 and BT549 cells. (E-F) MCF7 (E) and BT549 (F) cells with DTL or empty vectors were added 5 μM JNK-IN-8. Transwell and Matrigel assays showed that JNK inhibitor reduced the migration and invasion abilities of cancer cells. Statistical analysis results were shown in the right panel. *, P < 0.05, **, P < 0.01, NS, no significance based on the Student t test. All results are from three or four independent experiments. Error bars, SD. (TIF 7116 kb) [file 13046_2019_1358_MOESM8_ESM.tif]
