# Supplementary material for: DTL promotes cancer progression by PDCD4 ubiquitin-dependent degradation
Source: J Exp Clin Cancer Res. 2019 Aug 13;38:350. doi: 10.1186/s13046-019-1358-x (PMC6693180; doi:10.1186/s13046-019-1358-x)
Supplement: Supplementary file 9 — Table S1. The primer sequences used for plasmids construction were listed. (DOC 35 kb) [file 13046_2019_1358_MOESM9_ESM.doc]

| Primer Sequences for DTL and PDCD4 plasmids | |
| --- | --- |
| DTL 1-398aa | Forward: atgctcttcaattcggtgct  Reverse: tggtttctcctctaagcctc |
| DTL 1-498aa | Forward: atgctcttcaattcggtgct  Reverse: cttgaaagatgaaggtgg |
| DTL 1-598aa | Forward: atgctcttcaattcggtgct  Reverse: aaggtcttcctggttaccag |
| DTL | Forward: atgctcttcaattcggtgct  Reverse: taattctgttgagtgttcag |
| shDTL#1 | Forward: CCGGCCGAGGATGAATGCTGTGTTTCTCGAGAAACACAGCATTCATCCTCGGTTTTTG  Reverse:  AATTCAAAAACCGAGGATGAATGCTGTGTTTCTCGAGAAACACAGCATTCATCCTCGG |
| shDTL#2 | Forward: CCGGCCTGGTGAACTTAAACTTGTTCTCGAGAACAAGTTTAAGTTCACCAGGTTTTTG  Reverse: AATTCAAAAACCTGGTGAACTTAAACTTGTTCTCGAGAACAAGTTTAAGTTCACCAGG |
| shDTL#3 | Forward: CCGGGCCTAGTAACAGTAACGAGTACTCGAGTACTCGTTACTGTTACTAGGCTTTTTG  Reverse:  AATTCAAAAAGCCTAGTAACAGTAACGAGTACTCGAGTACTCGTTACTGTTACTAGGC |
| shPDCD4#1 | Forward:  CCGGTCTTACAGTCTTAGGTGTTACCTCGAGGTAACACCTAAGACTGTAAGATTTTTG  Reverse:  AATTCAAAAATCTTACAGTCTTAGGTGTTACCTCGAGGTAACACCTAAGACTGTAAGA |
| shPDCD4#2 | Forward:  CCGGGGGTGAGTGAAGCTACTAAACCTCGAGGTTTAGTAGCTTCACTCACCCTTTTTG  Reverse:  AATTCAAAAATCTTACAGTCTTAGGTGTTACCTCGAGGTAACACCTAAGACTGTAAGA |
| shPDCD4#3 | Forward:  CCGGTCCGCCGCCACGATTGGCCAGAGGCGGCGGTGCTAACCGGTCCTCGAGTTTTTG  Reverse:  AATTCAAAAACTTGCAGTCTTAGATGTTATACTCGAGTATAACATCTAAGACTGCAAG |
