# Supplementary material for: DTL promotes cancer progression by PDCD4 ubiquitin-dependent degradation
Source: J Exp Clin Cancer Res. 2019 Aug 13;38:350. doi: 10.1186/s13046-019-1358-x (PMC6693180; doi:10.1186/s13046-019-1358-x)
Supplement: Supplementary file 10 — Table S2. The antibodies used and their corresponding bands were listed. (DOC 33 kb) [file 13046_2019_1358_MOESM10_ESM.doc]

| Antibodies | Companies |
| --- | --- |
| DTL | Novus |
| PDCD4 | Cell Signaling Technology |
| JNK | Cell Signaling Technology |
| p-JNK | Cell Signaling Technology |
| Jun | Cell Signaling Technology |
| p-Jun | Cell Signaling Technology |
| FLAG flag | SIGMA-ALDRICH |
| HA flag | SIGMA-ALDRICH |
| MYC flag | Cell Signaling Technology |
| GAPDH | Abcam |
| β-actin | Zhong Shan Jin Qiao Biology |
| CUL4A | Cell Signaling Technology |
