# Supplementary material for: DTL promotes cancer progression by PDCD4 ubiquitin-dependent degradation
Source: J Exp Clin Cancer Res. 2019 Aug 13;38:350. doi: 10.1186/s13046-019-1358-x (PMC6693180; doi:10.1186/s13046-019-1358-x)
Supplement: Supplementary file 11 — Table S3. Primers for Quantitative real-time PCR were listed. (DOC 28 kb) [file 13046_2019_1358_MOESM11_ESM.doc]

| Primers for Quantitative real-time PCR | |
| --- | --- |
| DTL | Forward: ACCAGGAAGACCTTAGTA  Reverse: AAGCATACGGACTGATAG |
| GAPDH | Forward: AGGTGAAGGTCGGAGTCAAC  Reverse: CGCTCCTGGAAGATGGTGAT |
